# “Vaccine Diplomacy”: Historical Perspectives and Future Directions

**DOI:** 10.1371/journal.pntd.0002808

**Published:** 2014-06-26

**Authors:** Peter J. Hotez

**Affiliations:** 1 Sabin Vaccine Institute and Texas Children's Hospital Center for Vaccine Development, Departments of Pediatrics and Molecular Virology and Microbiology, National School of Tropical Medicine at Baylor College of Medicine, Houston, Texas, United States of America; 2 Departments of Medical Humanities and Biology, Baylor University, Waco, Texas, United States of America; 3 James A. Baker III Institute for Public Policy, Rice University, Houston, Texas, United States of America; Lindsley F. Kimball Research Institute, New York Blood Center, United States of America

*Vaccine diplomacy is the branch of global health diplomacy that relies on the use or delivery of vaccines, while vaccine science diplomacy is a unique hybrid of global health and science diplomacy. Both offer innovative opportunities to promote United States (US) foreign policy and diplomatic relations between adversarial nations. Vaccine science diplomacy could also lead to the development and testing of some highly innovative neglected disease vaccines.*

## Introduction: Origins and Definitions

International cooperation for purposes of infectious and tropical disease control goes back to at least the 14th century, when early concepts of quarantine were introduced in Dubrovnik on the Adriatic Coast of Croatia [1], [2], and to the later date of 1851, when Europe held its first International Sanitary Conference for multilateral cooperation to prevent the spread of cholera and, subsequently, plague and yellow fever [3]. Such efforts led to a series of international sanitary treaties and conventions and ultimately to the formation of the Pan American Health Organization and the later establishment of the World Health Organization (WHO) [3], [4].

Some scholars trace our current framework for global health diplomacy to the writings of Dr. Peter G. Bourne in his role as special assistant for health issues to US President Jimmy Carter [5] and later (during the first years of the 21st century) to the launch of the Millennium Development Goals (MDGs) and the release of the “Report of the Commission for Macroeconomics and Health”, when global health was placed squarely in the international diplomacy arena [6]. Among the driving forces for these activities was an urgent need for diplomatic collaboration to combat pandemics caused by HIV/AIDS and seasonal and avian influenza, which came with the revelation that such diseases are threats to economic development and both national security and foreign policy interests [7]. There were also practical considerations concerning potential bioterrorist threats and situations that required international diplomacy, such as when Indonesia balked at sharing its time-sensitive avian influenza data or when Nigeria and Pakistan halted polio and other immunization initiatives because of religious tensions [7]–[11].

In 2007, foreign ministers from seven countries—Brazil, France, Indonesia, Norway, Senegal, South Africa, and Thailand—issued the landmark “Oslo Ministerial Declaration” that formally linked global health to foreign policy [12]. At that time, Kickbusch et al. defined global health diplomacy in terms of processes by which governments and civil societies both “position health in foreign policy negotiations” and create new types of “global health governance” [13], [14]. More recently, Kickbusch and Lokeny defined it as a “system of organization and communications and negotiation processes that shape global policy environment in the sphere of health and its determinants” [15].

A key element of modern global health diplomacy is that “no longer do diplomats just talk to other diplomats”, but instead a variety of experts in different areas and disciplines are now brought in to solve timely global health issues [13]. Katz et al. [9] have since categorized different aspects of global health diplomacy to include the following: (1) core diplomacy, referring to “classical Westphalian negotiations” between nations leading to bilateral and multilateral treaties, such as the recent WHO Framework Convention on Tobacco Control and International Health Regulations (IHR) 2005; (2) multistakeholder diplomacy, i.e., negotiations between or among nations and international agencies such as WHO, the GAVI Alliance, United States Agency for International Development (USAID), and nongovernmental organizations (NGOs); and (3) informal diplomacy, which includes peer-to-peer scientific partnerships, private funders such as the Bill & Melinda Gates Foundation, and even some government employees from USAID or the US military working more or less independently in the field due to unique circumstances [9]. Michaud and Kates have identified similar forms of global health diplomacy [16].

Kickbusch and Lokeny have also noted recently that the WHO director-general made frequent mention of health diplomacy in her remarks at the January 2013 executive session [15]. Among the factors responsible for this emphasis are globalization associated with the renewed emphasis on “soft power”, security policy, trade agreements, and policies concerning the environment and international development, as well as the inclusion of health issues as part of the United Nations and summits held by various government organizations and agencies, such as the Group of Eight (G8) and Group of Twenty (G20) nations, the European Union (EU), the Organization of the Islamic Conference (OIC), and the BRICS (Brazil, Russia, India, China, and South Africa) countries [15]. Still another factor is the increasing use of health attachés embedded in foreign delegations and agencies and increasing dialogue with low- and middle-income countries [15]. With regards to the G20 (and their BRICS-country components), I introduced the term “blue marble health” to refer to the unexpectedly high neglected disease burden among the poor living in emerging economies and even some G20 countries, circumstances such that these nations could drastically reduce global burdens of neglected diseases by taking greater responsibility for their own health concerns [17], [18].

## Vaccine Diplomacy and Vaccine Science Diplomacy: Definitions

Beginning in 2001, the broad framework of global health diplomacy outlined above helped to generate the concepts of vaccine diplomacy and vaccine science diplomacy [19]–[24]. Vaccine diplomacy refers to almost any aspect of global health diplomacy that relies on the use or delivery of vaccines and encompasses the important work of the GAVI Alliance, as well as elements of the WHO, the Gates Foundation, and other important international organizations. Central to vaccine diplomacy is its potential as a humanitarian intervention and its proven role in mediating cessation of hostilities and even cease-fires during vaccination campaigns [20]–[22], [25]. In this case, the lead actor may come from an international organization, such as WHO or the United Nations Children's Fund (UNICEF), or an associated nongovernmental organization.

A subset of vaccine diplomacy is vaccine science diplomacy, which is a hybrid of elements of global health diplomacy and science diplomacy. I use the term “vaccine science diplomacy” narrowly to refer to the joint development of life-saving vaccines and related technologies, with the major actors typically scientists. Of particular interest, the scientists may be from two or more nations that often disagree ideologically or even from nations that are actively engaged in hostile actions. This definition is along the lines of what Katz et al. would call informal global health diplomacy based on peer-to-peer scientific interactions [9], together with elements of science diplomacy in which the representative nation projects power through its scientific prowess and reputation, as Abelson and others articulated for US science and applied technology during the Cold War [26]–[28] or more recently as can be seen in outreach to the Islamic world [29] and targeted initiatives for less developed countries [30]. Unlike many forms of global health diplomacy, this aspect of vaccine diplomacy is led by scientists.

An underlying theme of both vaccine and vaccine science diplomacies is that vaccines are unique in comparison to other medical or public health interventions. By some estimates, vaccines are the single most powerful intervention ever developed by humankind in terms of the lives that they save. By one estimate, modern vaccines have saved more lives than those that were lost in the world wars during the 20th century [21]–[23].

## The Historical Context

Both vaccine diplomacy and vaccine science diplomacy might be best understood by reviewing their historical successes (Table 1). Indeed, an interesting but little-known feature is how diplomacy is intimately tied to the initial development and delivery of many vaccines.

**Table 1:** Historical milestones in vaccine diplomacy.

Years	Specific Vaccine(s)	Actions	Reference
1800–1805	Smallpox	Edward Jenner promotes vaccine use in Russia, Turkey, and Spain and with Native Americans in the Spanish colonies of Mexico, the Five Nations of Canada, and the United States.	[31], [32]
1801	Smallpox	The chaplain of Congress, Dr. Edward Gantt, vaccinates Native American diplomats visiting Washington, D.C.	[32]
1803	Smallpox	The Lewis and Clark Expedition provides vaccine intended for Native Americans, but it is unclear if successful vaccinations were performed.	[32]
1803–1815	Smallpox	During the Napoleonic Wars, Jenner calls for prisoner release and other diplomatic functions. In a letter to the National Institute of France, he writes that “the sciences are never at war.”	[31]
1851	-	The First International Sanitary Conference is held in Europe.	[3]
1888	-	In a speech on the inauguration of the Pasteur Institute, Louis Pasteur states, “Science knows no country, because knowledge belongs to humanity and is the torch which illuminates the world.”	[31], [33]
1891–present	-	International network of Pasteur Institutes begins, initially in Saigon, for purposes of fundamental research and research on vaccines for rabies and other infectious diseases.	[34]
1892–1897	Cholera and plague	After first testing the vaccines on himself, Dr. Waldemar Haffkine travels to India to inoculate tens of thousands of people with his prototype cholera and plague vaccines.	[35]
1902	-	Formation of the International Sanitary Bureau (present-day Pan American Health Organization)	[4]
1946–48	-	Formation of the World Health Organization	[3]
1956–1959	Polio	Dr. Albert Sabin travels to the USSR and collaborates with Dr. Mikhail Chumakov, ultimately testing an oral vaccine on 10 million children and then on 100 million people under the age of 20.	[36]
1962–1966	Smallpox	The USSR provides 450 million doses of vaccine for an eradication campaign, while the US provides financial support.	[37]
1968	-	Formation of the Fogarty International Center of the NIH	
Mid-1970s		Formation of PATH	
1980s and 1990s	Polio and other vaccines	“Days of tranquility” for immunizations are held in more than a dozen war-torn countries.	[25]
1987	-	Indo-US Vaccine Action Program (VAP) is administered under the auspices of NIAID, NIH.	[38]
1990–91	-	Children's Vaccine Initiative (CVI)	
1993	-	Formation of the Sabin Vaccine Institute	[58]
	-	Formation of the Infectious Diseases Research Institute	
1997	-	Formation of the International Vaccine Institute	
1997	-	Formation of the Bill & Melinda Gates Foundation	
2000	-	GAVI Alliance is established, ultimately providing vaccines for North Korea.	[39], [41]
2001	-	“Vaccine diplomacy” enters the literature.	[19]
2007		Formation of program in Sustainable Immunization Financing at Sabin Vaccine Institute	[65]
2007	Influenza	Under the auspices of the WHO, Brazil, India, Indonesia, Mexico, Thailand, and Vietnam receive US and Japanese grants for influenza vaccine manufacturing capacity and technology transfer.	[52]
2008	Yellow Fever	Outbreak of urban yellow fever—the neighboring countries of Paraguay mobilize to ensure access to yellow fever vaccine.	[45]
2009	H1N1 Influenza A	Intergovernmental Meeting (IGM) on Pandemic Influenza Preparedness Framework for the Sharing of Influenza Viruses and Access to Vaccines and Other Benefits	[43]
2010	Cholera	Call for international cholera vaccine stockpile as a humanitarian and diplomatic resource	[44]
2011	-	Decade of Vaccines Collaboration	[46]
2012	-	The Global Vaccine Action Plan (GVAP)—endorsed by the 194 Member States of the World Health Assembly in May 2012	[47], [48]
2013	Leishmaniasis and other neglected tropical diseases	Joint statement on vaccine diplomacy between US and Iran	[54]
2013	-	State Department forms new Office of Global Health Diplomacy.	

The first vaccine discovered in modern times was in 1798 by Britain's Edward Jenner, who found that cowpox administered as an inoculum could prevent smallpox [31]; the term vaccine is derived from *vacca*, the Latin term for “cow”. Because smallpox produced such devastating and massive killer epidemics (especially among indigenous populations in the New World), the first vaccine almost immediately attained international acclaim in the first years of the 19th century [31], [32]. For example, from 1800 to 1805, Jenner corresponded widely and internationally and advised countries as diverse as Russia, Spain, and Turkey and Native American tribes and nations in Canada and Mexico on how to prepare and administer the smallpox vaccine [31], [32]. Among the earliest examples of vaccine diplomacy, in 1801 Dr. Edward Gantt, the chaplain of the US Congress, vaccinated Native American diplomats who were visiting Washington, D.C., and in 1803 the Lewis and Clark Expedition was provided smallpox vaccine intended for Native Americans living on the western frontier, although it is unclear if successful vaccinations were actually performed [32]. From 1803 to 1815 during the Napoleonic wars between England and France, Jenner himself was called on for diplomatic functions, including prisoner releases [31]. Jenner was honored in France and wrote in a letter to the National Institute of France that “the sciences are never at war,” while Napoleon was supposed to have once stated, “Jenner—we can't refuse that man anything” [19], [31].

The next set of vaccines, including a new rabies vaccine, was developed almost one hundred years later by France's Louis Pasteur. In a speech at the inauguration of his institute in Paris in 1888, Pasteur stated that “science knows no country, because knowledge belongs to humanity and is the torch which illuminates the world” [31], [33]. Before the close of the century, scientists from the Pasteur Institute spread out to create a network of laboratories in Francophone countries in Indochina (beginning with the Saigon Pasteur Institute [1891]) and North Africa [34], especially for the preparation and administration of rabies vaccine. Around this time (from 1892–1897), Dr. Waldemar Haffkine, a Jewish scientist from Ukraine working in France and Switzerland, traveled to India in order to inoculate tens of thousands of people with his prototype cholera and plague vaccines, but he did so only after first testing the vaccines on himself [35]. Today, the Haffkine Institute in Mumbai is an important microbiology research institute.

Vaccine science diplomacy entered its golden age during the Cold War between the US and the Union of Soviet Socialist Republics (USSR). Between 1956 and 1959, Dr. Albert Sabin from the US traveled to the USSR and collaborated with his Soviet virology counterparts, including Dr. Mikhail Chumakov, to develop a prototype oral polio vaccine and test it on 10 million Soviet children and ultimately 100 million people under the age of 20 [36]. The success of the collaboration depended on each scientist going to great lengths to convince their diplomatic liaisons to put aside ideologies for purposes of joint scientific cooperation [19]–[23], [36]. Today, the oral polio vaccine is leading to global eradication efforts. Similarly, between 1962 and 1966, the USSR pioneered a freeze-drying technique for smallpox vaccine and provided 450 million doses of vaccine to support global smallpox eradication campaigns in developing countries, while the US provided key financial support [37]. Such international collaborative efforts led to the global eradication of smallpox by the late 1970s, an effort led by Dr. D. A. Henderson [37]. Later, in the 1980s and following the visit of US Nobel Laureate Fred Robbins to India, the Indo-US Vaccine Action Program (VAP) was established to foster international collaboration in the areas of epidemiology, laboratory investigation, and vaccine clinical trials, quality control, and delivery [38]. VAP is maintained under the auspices of the National Institute of Allergy and Infectious Diseases of the US National Institutes of Health (NIH) [38]. In 1990–91, a Children's Vaccine Initiative was launched as an early attempt at global governance for developing pediatric vaccines for developing countries.

Vaccine diplomacy also flourished in the later decades of the 20th century. According to WHO's Health as a Bridge to Peace—Humanitarian Cease-Fires Project (HCFP), vaccines and vaccinations were used to negotiate so-called “days of tranquility” in more than a dozen countries during the 1980s and 1990s, including Afghanistan, Angola, Chechnya, Democratic Republic of Congo, El Salvador, Guinea Bissau, Iraq, Lebanon, Philippines, Sierra Leone, Sri Lanka, and Sudan [25].

## Modern Day Vaccine and Vaccine Science Diplomacy

Beginning in 2000, vaccines became integrated as key tools in helping developing nations achieve their MDGs and targets. Following the launch of the GAVI Alliance, many developing countries for the first time gained access to vaccines for combating rotavirus and *Haemophilus influenzae* type b (Hib), and a new vaccine for pneumococcal vaccine was developed [39], [40]. Partly because of these interventions, child mortality was reduced by almost one-half [40]. Included among these activities was GAVI's important work in providing vaccines for North Korea and other fragile states [41].

Among the initiatives relevant to vaccine diplomacy in the 21st century are international efforts to ensure universal or equitable access for low- and middle-income countries to urgently needed vaccines for diseases of pandemic potential. It was noted that many developing countries were on the “outside looking in” when it came to having access to influenza vaccines, including the vaccine for the H1N1 pandemic influenza in 2009 and prototype H5N1 avian influenza vaccines [42], [43]. As a result, Indonesia went through a period in which it refused to share timely influenza surveillance data with the WHO [42]. It was noted that IHR 2005 did not adequately spell out provisions on providing equitable access for vaccines [43], and it was probably not intended for this purpose. In 2009, an Intergovernmental Meeting (IGM) was held on pandemic influenza preparedness as a means to establish a framework for sharing influenza and other vaccines with developing countries [43]. Issues of developing country access again arose when cholera emerged in sub-Saharan Africa and Haiti; there was no mechanism to rapidly mobilize cholera vaccine, and calls went out to stockpile cholera vaccine as a humanitarian and diplomatic resource [44]. Also, in 2008 when yellow fever vaccine supplies were depleted during the first urban yellow fever outbreak in the Americas in decades, countries neighboring Paraguay helped to ensure that the vaccine was made available in that country [45]. In 2012, following the earlier launch of the Decade of Vaccines Collaboration [46], the Global Vaccine Action Plan (GVAP) was endorsed by the 194 Member States of the World Health Assembly as “a framework to prevent millions of deaths by 2020 through more equitable access to existing vaccines for people in all communities” [47]. A World Health Assembly resolution was adopted that recognizes access to vaccines as a fundamental right to human health [48]. The diplomatic community was also called on to address critical issues of noncompliance for polio and other vaccines intended for vulnerable populations living in Islamic countries. In 2003, a boycott of polio vaccinations in three northern Nigerian states from fears that the vaccine was contaminated with antifertility drugs (in order to sterilize Muslim girls) necessitated diplomatic intervention from the Government of Malaysia and the OIC [49]. Similar interventions are now required in Pakistan, where the Taliban and other extremist groups have assassinated vaccinators and other aid workers [50]. Some assassinations may have been carried out in retaliation for the Central Intelligence Agency (CIA)'s alleged role in establishing a fake vaccination campaign in Abbottabad, Pakistan, as a ruse in order to confirm the identity of members of Osama bin Laden's family [51]. Such activities represent a significant setback to vaccine diplomacy.

Of relevance to both vaccine and vaccine science diplomacy, in 2007 under the auspices of the WHO and the Global Pandemic Influenza Action Plan, six countries—Brazil, India, Indonesia, Mexico, Thailand, and Vietnam—received grants from the US and Japanese governments to establish in-country manufacturing capacity for influenza vaccines [52].

## Future Directions and Moving towards a Framework

While the historical and modern-day track records of vaccine and vaccine science diplomacy are impressive, they have not yet led to an overarching framework for its expanded role in foreign policy. Establishing such a framework might be especially useful for US foreign policy.

In 2009, President Obama traveled to Cairo where he spoke out about engaging scientists in the Muslim world and extending a hand in science diplomacy [53]. Despite the establishment of a valuable US Science Envoy program, to date such activities have not led to substantive joint vaccine partnerships despite the observation that several Islamic countries in the Middle East and Asia, including Egypt, Indonesia, Iran, and Saudi Arabia, have some capacity for vaccine product development [23]. With an Iranian scientist from the Tehran University of Medical Sciences, Dr. Mohammed Rokni, I recently advocated launching such efforts between the US and Iran and provided as an example the opportunity for developing a vaccine for leishmaniasis, which has devastated areas of conflict in the Middle East and North Africa [54]. Similar opportunities exist in order to partner with nations such as Cuba, which has considerable technical expertise both in producing and delivering vaccine [55], and possibly even countries such as North Korea, which has some technical capabilities [56].

**Figure 1:**
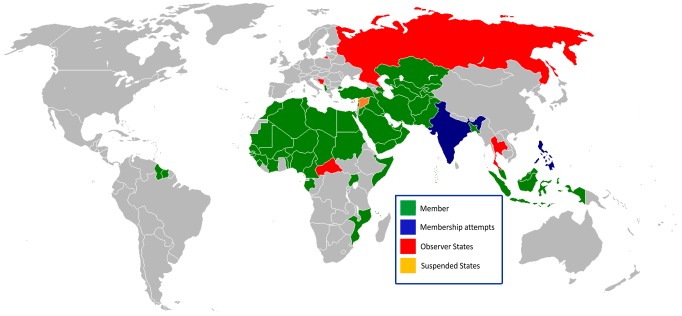
The OIC member nations. Figure adapted from Wikipedia: http://en.wikipedia.org/wiki/File:OIC_map.png.

Our Sabin Vaccine Institute and Texas Children's Hospital Center for Vaccine Development (Sabin), a nonprofit product development partnership (PDP) that uses industry practices to develop and test neglected disease vaccines, could occupy a key niche in vaccine diplomacy. Sabin's vaccine portfolio targets neglected tropical diseases (NTDs) that specifically affect the poorest people living in low- and middle-income countries. Because NTDs have been shown to promote poverty through their adverse effects on worker productivity, the health of girls and women, and child development, the vaccines under development at Sabin are sometimes referred to as the “antipoverty vaccines” [57], [58]. Moreover, most of the diseases targeted by the Sabin portfolio of vaccines occur in countries of direct relevance to vaccine diplomacy (Table 2) [59]. For example, more than one-third of the world's cases of hookworm infection, ascariasis, and trichuriasis occur in nations of the OIC, i.e., the world's Muslim countries (Figure 1), while almost one-half of the cases of schistosomiasis occur among the OIC countries [59]. Furthermore, both cutaneous and visceral leishmaniasis have emerged as the most significant infections arising in settings of ongoing conflict, with the former affecting hundreds of thousands of people in Syria and Syrian refugees, while the latter was the leading killer in the war between northern and southern Sudan during the 1980s and 1990s [60]. Some of these diseases are also widespread in some Latin American countries where leaders have expressed varying degrees of anti-American sentiment. While Sabin is currently conducting joint vaccine development with public-sector vaccine manufacturers in Brazil and Mexico, it is ready to embark on joint vaccine development with countries such as Cuba, Indonesia, and Iran, i.e., nations with either strained or even overtly hostile foreign relations with the US in past and recent years. As a form of projecting soft power with both allies and potential adversaries, such activities are consistent with what former Secretary Hillary Clinton termed “civilian power” [24].

**Table 2:** Sabin PDP vaccines under development of potential relevance to US foreign policy interests.

Disease Targeted (Approximate Number of People Affected)	Affected Geographic Areas of Interest to US Foreign Policy Interests	Stage of Development
Human hookworm infection (400 million)	OIC countries in Africa, the Middle East, and Asia	Phase 1
	India and China	
Schistosomiasis (250 million)	OIC countries in Africa and the Middle East	Completed current good manufacturing practice (cGMP) manufacture
Ascariasis and Trichuriasis (>800 million)	OIC countries in Africa, the Middle East, and Asia	Preclinical
	India and China	
Leishmaniasis (10 million)	Areas of conflict in the Middle East and North Africa, including OIC countries	Preclinical
Chagas disease (7–8 million)	Venezuela, Ecuador, Bolivia	Preclinical
SARS (None currently)	China	Preclinical

Beyond US foreign relations, there are opportunities for vaccines to promote cooperation between Asian nations. For instance, each of the largest Asian countries, i.e., China, India, Indonesia, Japan, and Vietnam, has capabilities to develop and produce new vaccines [56], [61]. China and India engaged in overt hostilities in 1964, while China's recent territorial claims in the East China Sea have sparked fresh tensions in the region [61]. Both Sabin and another PDP, the International Vaccine Institute (IVI) based in Seoul, Korea [62], could help mediate vaccine diplomacy between these nations. In addition, Brazil, which also has major vaccine capabilities, has initiated South-South partnerships with Lusophone Africa and could become an important actor in vaccine diplomacy [63]. Vaccine manufacturing organizations associated with many of the key OIC and Asian nations targeted for vaccine science diplomacy belong to the unique Developing Countries Vaccine Manufacturers Network (DCVMN) [64]. Both the GAVI Alliance and WHO could have key roles in coordinating these activities. These organizations also have a key role in a new Sustainable Immunization Financing program inaugurated with Gates Foundation support by Dr. Ciro De Quadros at the Sabin Vaccine Institute, which focuses on 12 African countries, five Asian countries, and one Central Asian country [65].

Today, the Division of International Relations of the NIH's Fogarty International Center maintains an important role in promoting international agreements between the US and governments throughout the world [66]. In the coming years, vaccine and vaccine science diplomacy activities could become incorporated into the new US State Department Office of Global Health Diplomacy [67], as well as into the WHO and its regional offices and within organizations such as the Bill & Melinda Gates Foundation and the Carlos Slim Health Institute. The power of vaccine and vaccine science diplomacy has been underexplored despite a noble track record that included promoting peace between the Cold War powers of the 1950s and 1960s, which also led to the development, testing, and delivery of two of the most important 20th century health interventions, i.e., the freeze-dried smallpox vaccine and oral polio vaccine, and the resulting global eradication of smallpox and near elimination of polio. The historical lessons from these accomplishments still have critical relevance to global health and blue marble health.

**Box 1. Potential Sites for Vaccine Diplomacy and US Foreign Policy**

- Sabin Vaccine Institute and Texas Children's Hospital Center for Vaccine Development, Houston, Texas, United States of America
- International Vaccine Institute, Seoul, Korea
- IDRI (Infectious Disease Research Institute), Seattle, Washington, United States of America
- PATH Vaccine Development Global Program, Washington, D.C., United States of America
- Finlay Institute, Havana, Cuba
- Birmex, Mexico, D.F., Mexico
- FIOCRUZ Bio-Manguinhos, Rio de Janeiro, Brazil
- Instituto Butantan, Sao Paulo, Brazil
- Vacsera, Cairo, Egypt
- Razi Vaccine and Serum Institute and Institut Pasteur, Tehran, Iran
- Biopharma, Bandang, Indonesia
